# Novel T cells with improved *in vivo* anti-tumor activity generated by RNA electroporation

**DOI:** 10.1007/s13238-017-0422-6

**Published:** 2017-05-18

**Authors:** Xiaojun Liu, Shuguang Jiang, Chongyun Fang, Hua Li, Xuhua Zhang, Fuqin Zhang, Carl H. June, Yangbing Zhao

**Affiliations:** 1 Key Laboratory for Experimental Teratology of Ministry of Education and Department of Immunology, School of Basic Medical Sciences, Shandong University, Jinan, 250012 China; 2 Center for Cellular Immunotherapies, University of Pennsylvania Cancer Center, Philadelphia, PA 19104 USA; 3 Department of Pathology and Laboratory Medicine, Perelman School of Medicine, University of Pennsylvania, Philadelphia, PA 19104-5156 USA

**Keywords:** T lymphocytes, CAR, manufacture, gene transfer, RNA electroporation

## Abstract

The generation of T cells with maximal anti-tumor activities will significantly impact the field of T-cell-based adoptive immunotherapy. In this report, we found that OKT3/IL-2-stimulated T cells were phenotypically more heterogeneous, with enhanced anti-tumor activity *in vitro* and when locally administered in a solid tumor mouse model. To further improve the OKT3/IL-2-based T cell manufacturing procedure, we developed a novel T cell stimulation and expansion method in which peripheral blood mononuclear cells were electroporated with mRNA encoding a chimeric membrane protein consisting of a single-chain variable fragment against CD3 and the intracellular domains of CD28 and 4-1BB (OKT3-28BB). The expanded T cells were phenotypically and functionally similar to T cells expanded by OKT3/IL-2. Moreover, co-electroporation of CD86 and 4-1BBL could further change the phenotype and enhance the *in vivo* anti-tumor activity. Although T cells expanded by the co-electroporation of OKT3-28BB with CD86 and 4-1BBL showed an increased central memory phenotype, the T cells still maintained tumor lytic activities as potent as those of OKT3/IL-2 or OKT3-28BB-stimulated T cells. In different tumor mouse models, T cells expanded by OKT3-28BB RNA electroporation showed anti-tumor activities superior to those of OKT3/IL-2 T cells. Hence, T cells with both a less differentiated phenotype and potent tumor killing ability can be generated by RNA electroporation, and this T cell manufacturing procedure can be further optimized by simply co-delivering other splices of RNA, thus providing a simple and cost-effective method for generating high-quality T cells for adoptive immunotherapy.

**Electronic supplementary material:**

The online version of this article (doi:10.1007/s13238-017-0422-6) contains supplementary material, which is available to authorized users.

## INTRODUCTION

T lymphocytes can be modified by gene transfer to enhance their specific anti-tumor activities for cancer treatment (Brentjens et al., 2013; Lee et al., 2015; Morgan et al., 2006; Porter et al., 2011). To further improve this therapeutic approach, efforts are underway to define and generate better T cells. In general, T lymphocytes must be expanded to sufficient quantities before use. Several *ex vivo* cell manufacturing platforms can be used to produce clinical-grade products with large numbers of T cells for adoptive immunotherapy trials. These approaches include the use of anti-CD3/CD28 beads (Levine et al., 1997), the direct addition of anti-CD3 antibodies to peripheral blood mononuclear cells (PBMCs) in the presence of IL-2 (OKT3/IL-2) (Riddell and Greenberg, 1990) and cell-based artificial APCs (Suhoski et al., 2007). T cells generated by different methods have different phenotypes and *in vitro*/*in vivo* functions. The development of manufacturing strategies to generate T cells with maximal anti-tumor activities *in vivo* will significantly impact T-cell-based adoptive immunotherapy. All current T cell manufacturing procedures require antibodies, which are limiting factors and potential impediments due to both their cost and supply when large quantities of expanded T cells are required. Moreover, the mouse origin of the antibodies may be carried over to the T cell products, potentially rendering them immunogenic and thereby limiting the therapeutic efficacy of the infused T cells. In our previous report, a comparison of T cells generated from two methods commonly used in clinical trials showed that compared with OKT3/IL-2-stimulated T cells, CD3/CD28-Dynabead-stimulated T cells were more uniformly central memory cells with a significantly potent ability to control leukemia in Nalm6 mice model following intravenous infusion (Barrett et al., 2014). In our current study, intraperitoneal injection of mesothelin CAR RNA-electroporated T cells generated by OKT3/IL-2 stimulation achieved a rapid and sustained reduction in disease burden than those generated using CD3/CD28 Dynabead against intraperitoneal human-derived mesothelioma tumors that had grown in mice for 56 days before treatment (Campagnolo et al., 2004; Zhao et al., 2010). Furthermore, we found that T cells could be efficiently stimulated and expanded by direct electroporation of PBMCs with mRNA encoding a chimeric membrane protein consisting of a single-chain variable fragment (scFv) against CD3 (OKT3) and the intracellular domains of CD28 and 4-1BB (OKT3-28BB) in the presence of IL-2. We also found that co-electroporation with other RNA molecules, such as CD86 and 4-1BBL, can further change the phenotype and function of OKT3-28BB RNA-electroporated T cells (RNA-T cells). Interestingly, T cells expanded by co-electroporation of OKT3-28BB with CD86 and 4-1BBL showed less differentiated phenotypes, although they still maintained a tumor lytic ability as potent as that of OKT3/IL-2-stimulated T cells. In different tumor mouse models, T cells expanded from OKT3-28BB/CD86/4-1BBL RNA electroporation showed anti-tumor activities superior to those of OKT3/IL-2 T cells and similar to those of CD3/CD28 Dynabead T cells. Hence, T cells with both a young phenotype and potent killing ability can be generated by RNA electroporation, and this T cell manufacturing procedure can be potentially further optimized by simply co-delivering other splices of RNA.

## RESULTS

### RNA CAR-transferred T cells expanded via OKT3/IL-2 were heterogeneous in phenotype and had enhanced and persistent function *in vitro*

**Figure 1:**
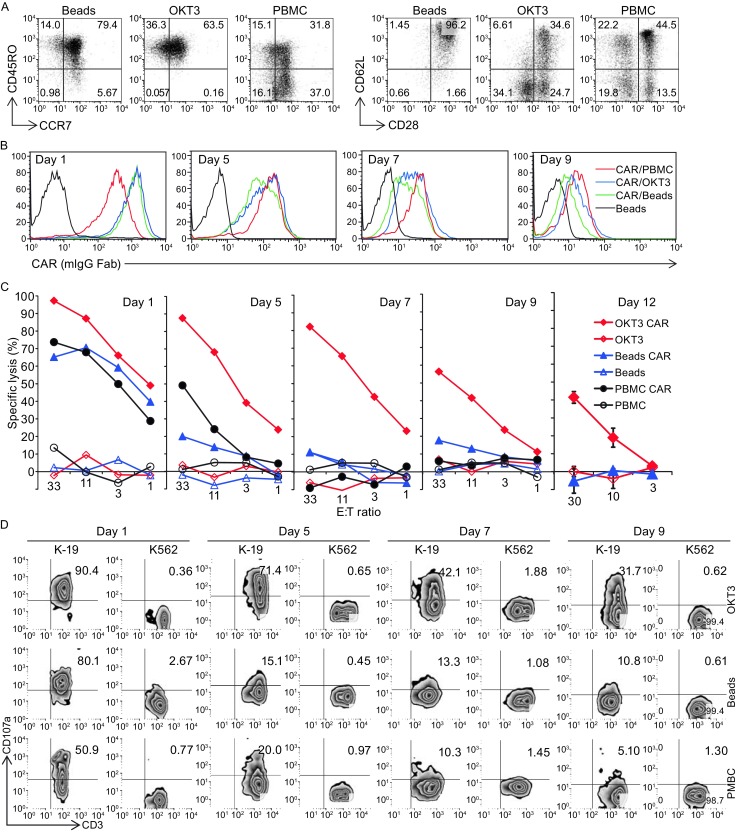

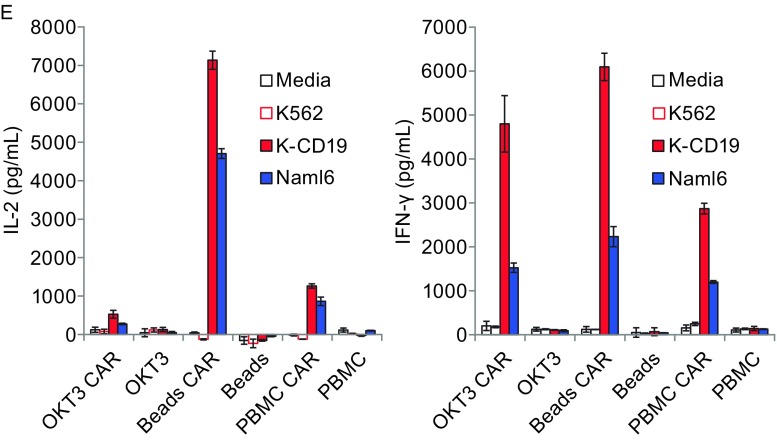
***In vitro*** **function comparison of CD3/CD28 Dynabead-stimulated (Beads) and OKT3/IL-2-stimulated (OKT3) T cells**. (A) Phenotype of the expanded T cells, compared with T cells from non-stimulated PBMCs. (B) CAR expression of CD19-BBZ CAR RNA-transferred T cells at different time points post-RNA electroporation. (C) Lytic activity against Nalm6 in a 4-h flow cytometry-based CTL assay for CAR RNA-transferred T cells at different time points post-RNA electroporation. (D) CD107a detection of T cells stimulated by K562-CD19 (K-19) at different time points post-RNA electroporation. (E) Cytokine production of CD19-BBZ CAR RNA-transferred T cells

**Figure 2:**
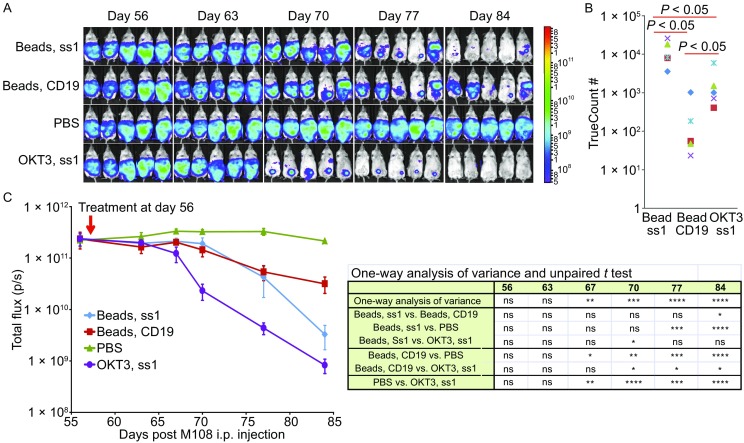
**Treatment of advanced vascularized tumors in mice with RNA CAR T cells**. (A) Visual representation of disease burden in mice with an M108 tumor treated with expanded T cells. Disseminated intraperitoneal (i.p.) tumors were established in NSG mice (*n* = 5 per group) via injection with 8 × 10^6^ M108-Luc cells. Beginning on day 56, a single dose of RNA CAR-electroporated T cells (2.5 × 10^7^) was injected (i.p.). (B) Detection of human T cells in the peripheral blood of treated mice 40 days post-treatment. Human CD45^+^ cells were detected using a Trucount flow assay. (C) BLI data and statistical analysis for the experiment. Bars, SE. A greater reduction in disease burden was noted in the mice treated with T cells expanded with OKT3 and expressing ss1BBZ CAR compared with all other groups

By comparing the phenotype of the expanded CD3/CD28 Dynabead T cells and the OKT3/IL-2 T cells, it was found that the CD3/CD28 Dynabead T cells had a larger central memory population, as evidenced by 79.4% CD45RO^+^/CCR7^+^ (versus 63.5% for OKT3 T cells, *P* < 0.01) with a uniformly younger phenotype (96.2% CD62L^+^/CD28^+^ versus 34.6% for OKT3 T cells, *P* < 0.01) (Fig. 1A). The T cells were transiently transferred with a CD19-BBZ CAR by RNA electroporation, and although the CAR expression levels at different time points were similar (Fig. 1B), the OKT3/IL-2 T cells demonstrated increased and sustained antigen-specific lytic activities, as evidenced by both a 4-h CTL killing assay and a CD107a assay, compared with the CD3/CD28 Dynabead T cells (Fig. 1C and 1D). After stimulation by CD19 positive leukemia lines, the CD3/CD28 Dynabead T cells and OKT3/IL-2 T cells secreted similar amounts of IFN-gamma. However, the OKT3/IL-2 T cells produced significantly lower amounts of IL-2. This decreased IL-2 production and increased lytic activity in the OKT3/IL-2 T cells suggests that the OKT3/IL-2 T cells are more differentiated toward effector memory cells, with increased direct tumor-controlling ability and decreased migration and proliferation potential. This finding was supported by our previous report on xenograft models of Nalm6 leukemia, in which CD3/CD28 Dynabead T cells proliferated earlier and to a greater extent than did OKT3/IL-2 T cells, resulting in better disease control. This phenomenon occurred because the Dynabead T cells had a younger phenotype or higher proliferative capacity (Barrett et al., 2014). The finding that OKT3/IL-2 T cells have an more differentiated phenotype and an increased *in vitro* lytic ability suggests that these T cells may have more potent anti-tumor activity if they are locally administered, such as in a situation where direct anti-tumor potency is more important than migration and proliferation. As shown in Figure 2A and 2C, in an intraperitoneal mesothelioma mouse model, a single dose of 25 million CAR RNA-electroporated T cells was injected intraperitoneally 56 days post-tumor inoculation. A rapid and greater reduction in disease burden was noted in the mice treated with ss1BBZ CAR T cells expanded with OKT3/IL-2 compared with ss1BBZ CAR T cells expanded with CD3/CD28 Dynabead. Due to the development of non-specific T cell/tumor alloreactivity and severe GVHD (data not shown), the experiment was terminated on day 84 post-tumor inoculation. Human T cells (hCD45^+^/hCD3^+^) in the peripheral blood of the treated mice were measured using a Trucount flow assay, and there were significantly more T cells in the mice treated with ss1.BBZ CD3/CD28 Dynabead T cells than in those treated with either the control CD19.BBZ CD3/CD28 bead T cells or the ss1.BBZ OKT3/IL-2 T cells (Fig. 2B).

### T cells can be stimulated and expanded by electroporating mRNA encoding a chimeric membrane protein consisting of an scFv against CD3 from OKT3 and the intracellular domains of CD28 and 4-1BB

**Figure 3:**
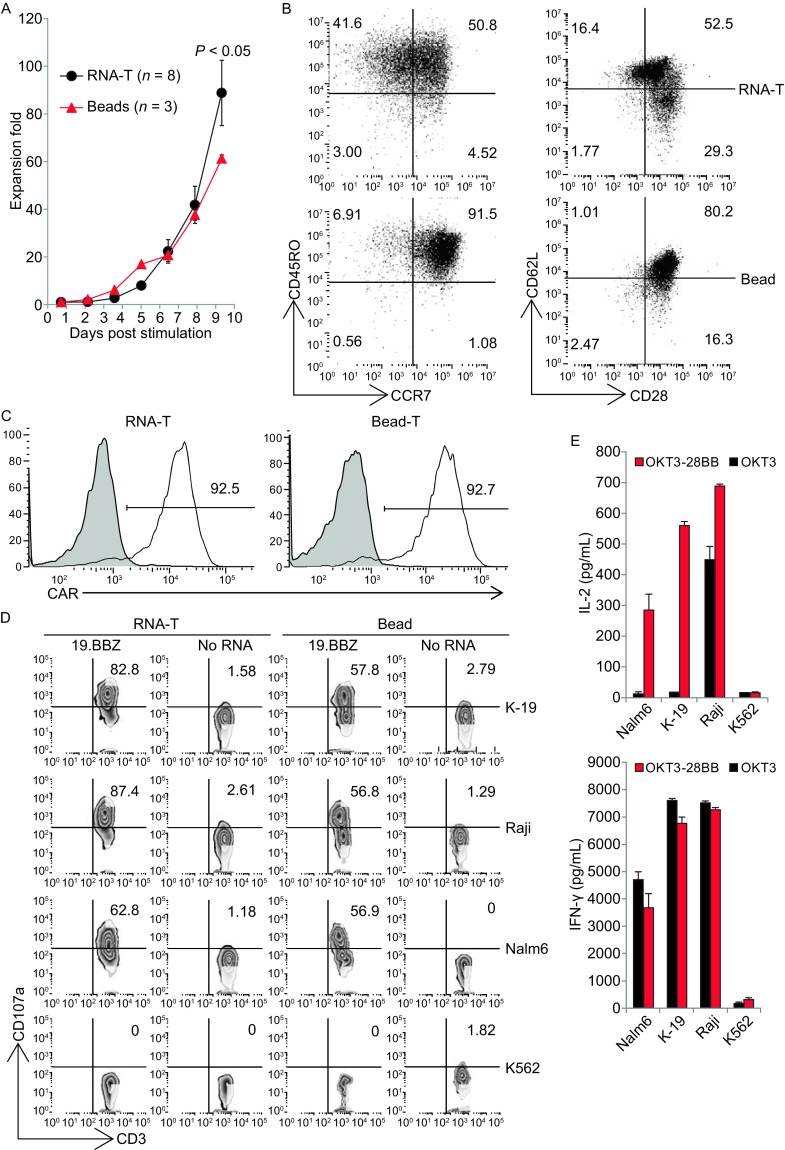
***In vitro*** **function of RNA-T cells**. (A) Expansion of T cells generated using CD3/CD28 Dynabead (Beads) and OKT3-28BB RNA electroporation (RNA-T). (B) Phenotype of the expanded T cells. Flow cytometry results of day-9 expanded T cells. (C) CAR expression of CD19-BBZ CAR RNA-transferred T cells 24 h post-electroporation. (D) Flow cytometry detection of CD107a for T cells stimulated by CD19-positive cell lines K562-CD19 (K-19), Raji or Nalm6. (E) Cytokine secretion of the T cells detected by ELISA. CD19-BBZ CAR RNA-transferred T cells were stimulated by CD19-positive cell lines K562-CD19 (K-19), Raji or Nalm6 for 24 h, and the supernatant was harvested for cytokine detection

To further improve the T cell manufacturing procedure, we developed a novel T cell stimulation and expansion method in which PBMCs were electroporated with RNA encoding a chimeric membrane protein consisting of an scFv against CD3 and the intracellular domains of CD28 and 4-1BB (OKT3-28BB). Activation and expansion methods for most T cells require OKT3, a CD3 antibody, which is of mouse origin and can be carried into the T cell products. The mouse Ab carried over to the T cells could potentially cause an immunoreaction against the infused T cells, thus negatively affecting the treatment. PBMCs from healthy donors were electroporated with *in vitro* transcribed RNA encoding OKT3-28BB and the introduced OKT3-28BB can be detected on the cell surface 18 h after electroporation (Fig. S2). The cells were cultured for T cell expansion and compared with CD3/CD28 Dynabead-stimulated T cells. T cells stimulated by the electroporation of OKT3-28BB RNA (RNA-T cells) expanded as efficiently as the CD3/CD28 Dynabead T cells (Fig. 3A). Phenotype analysis confirmed that the CD3/CD28 Dynabead T cells were uniformly central memory T cells (91.5% CD45RO/CCR7 double-positive), with 80.2% of the cells expressing CD62L and CD28. However, the RNA-T cells were more heterogeneous; they had a more differentiated phenotype (50.8% CD45RO/CCR7 double-positive), and 52.5% of the cells expressed CD62L and CD28 (Fig. 3B). Except significantly high expression of 2B4 in RNA-T cells, both CD3/CD28 Dynabead T cells and RNA-T cells expressed CTLA4, TIM-3, LAG-3, and PD-1 at the same levels (Fig. S3). Their functions were tested via stimulation with the CD19-positive cell lines K562-CD19, Raji or Nalm6 after the expanded T cells were transferred with CD19-BBZ CAR RNA. CAR was expressed at similar levels in both the CD3/CD28 Dynabead T cells and RNA-T cells (Fig. 3C), while CD107a was more highly expressed in the RNA-T cells than in the CD3/CD28 Dynabead T cells (Fig. 3D). The heterogeneous phenotype and increased *in vitro* lytic activity of the RNA-T cells relative to the CD3/CD28 Dynabead T cells indicate that the RNA-T cells are similar to OKT3/IL-2 T cells. However, although the levels of IFN-γ secreted by the RNA-T cells and OKT3/IL-2 T cells were comparable, IL-2 production was much higher in the RNA-T cells than in the OKT3 T cells after electroporation with CD19-BBZ CAR RNA and stimulation with the CD19-positive cell lines Nalm6, K562-CD19, and Raji (Fig. 3E).

### Less differentiated T cells with potent killing ability can be generated by co-electroporation of RNAs encoding OKT3-28BB and co-stimulatory molecules

**Figure 4:**
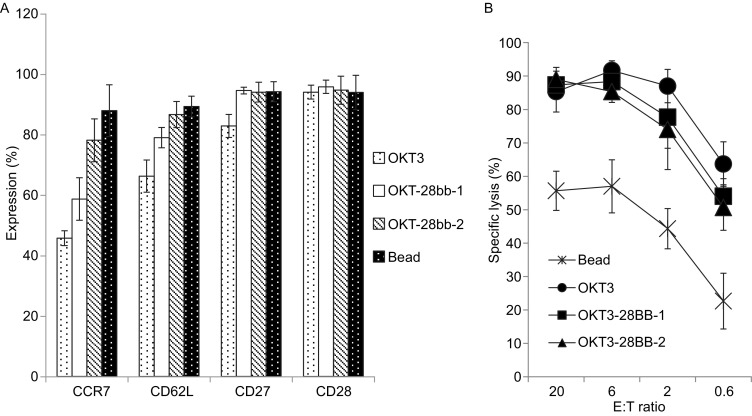
**T cells with a less differentiated phenotype and potent lytic activity were expanded by RNA co-electroporation**. (A) Detection of less differentiated T cell markers using flow cytometry for T cells generated by OKT3/IL-2 (OKT3), OKT3-28BBZ RNA electroporation (OKT-28bb-1) or OKT3-28BBZ, CD86 and 4-1BBL RNA co-electroporation (OKT-28bb-2). (B) Four-hour flow-based CTL assay of CD19.BBZ CAR RNA-electroporated T cells

We next examined whether the co-electroporation of co-stimulatory molecular ligand RNA for CD86 and 4-1BBL, which provide additional costimulatory signals through T cells’ native receptor CD28 and 4-1BB respectively, could further change the phenotype and function of RNA-T cells. T cells co-electroporated with OKT3-28BB, CD86, and 4-1BBL (OKT3-28BB-86BBL) expressed CCR7 and CD62L at levels as high as those in CD3/CD28 Dynabead T cells, which was in contrast to the OKT3-28BB RNA-T cells and OKT3/IL-2 T cells (Fig. 4A). Interestingly, although the OKT3-28BB-86BBL RNA-T cells were primarily central memory T cells, their killing ability was still as strong as that of the OKT3/IL-2 T cells and OKT3-28BB RNA-T cells and was significantly stronger than that of the CD3/CD28 Dynabead-stimulated T cells (Fig. 4B). Hence, T cells with both a less differentiated phenotype and a potent killing ability can be generated by co-electroporation of RNAs encoding OKT3-28BB and the ligands of co-stimulatory molecules, CD86 and 4-1BBL.

### Potent *in vivo* anti-tumor activities of RNA electroporated or lentiviral-transduced RNA-T cells

**Figure 5:**
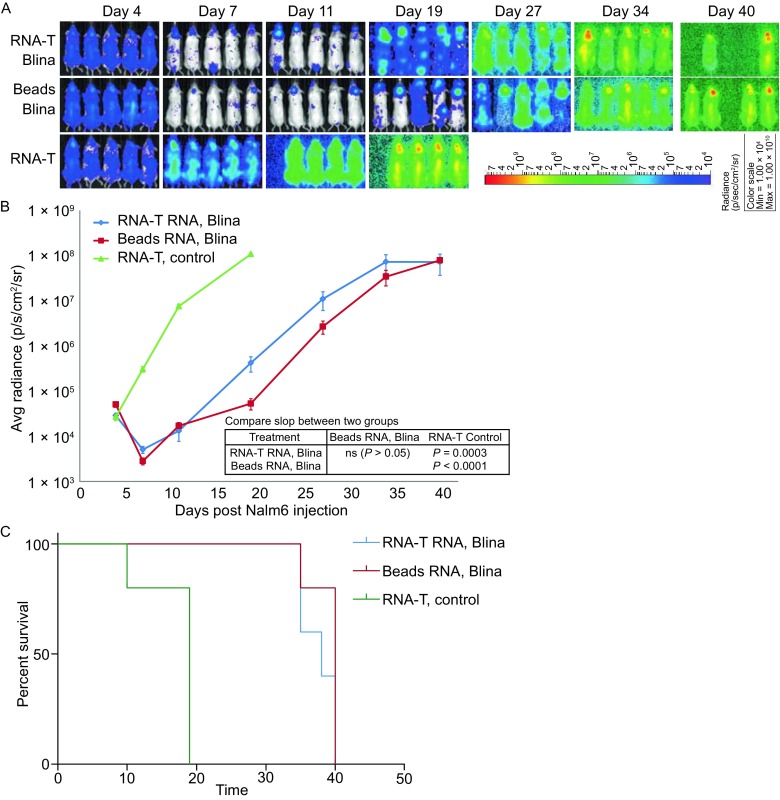
**Treatment of Nalm6 leukemia in mice with T cells transferred with RNA encoding CD19-CD3 BiTEs**. (A) Leukemia was established in NSG mice (*n* = 5 per group) by intravenous (i.v.) injection with 5 × 10^6^ Nalm6-CBG cells. Beginning on day 5, a single dose of 1 × 10^7^ CD19-CD3 BiTE (Blina) RNA-electroporated CD3/CD28 Dynabead T cells (Beads Blina) or RNA-T cells (RNA-T Blina) was injected (i.v.), using non-RNA-electroporated RNA-T cells as controls (RNA-T, control). (B) BLI was conducted at the time indicated. (C) Survival curve of the experiment

**Figure 6:**
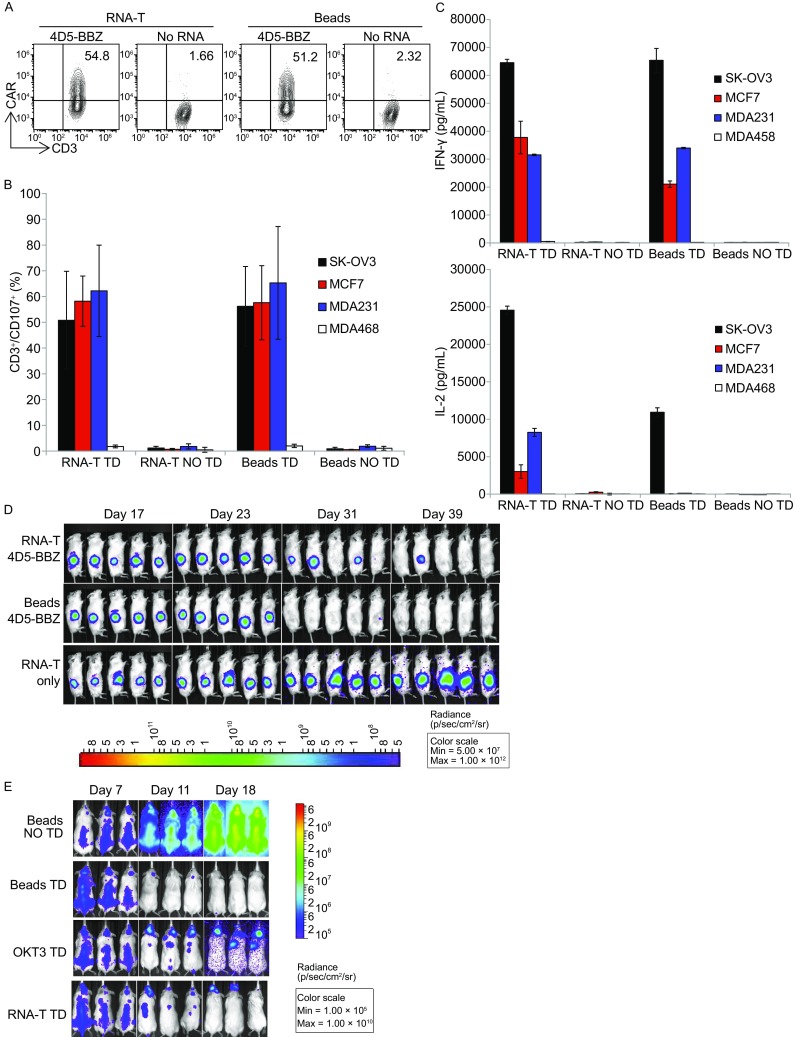
**Anti-tumor activities of lentiviral-transduced T cells tested** ***in vitro*** **and in mouse tumor models**. (A) CAR expression of RNA-T cells (RNA-T) or CD3/CD28 Dynabead T cells (Beads) lentiviral-transduced with an ErbB2 CAR (4D5-BBZ). (B) Summary of three independent experiments for detection of CD107a in 4D5-BBZ CAR transduced (TD) T cells stimulated by ErbB2-positive tumor lines SK-OV3, MCF7, and MDA231, using the tumor line MDA468 and non-transduced (NO TD) T cells as negative controls. (C) Cytokine production of T cells stimulated by ErbB2-positive tumor lines SK-OV3, MCF7, and MDA231 and an ErbB2-negative tumor line MDA468. (D) Mice (*n* = 5) were subcutaneously implanted with SK-OV3-CBG tumor cells (5 × 10^6^ cells/mouse) on the flank. The mice were treated with T cells (i.v.) on day 18 after SK-OV3-CBG tumor inoculation. A dose of 1 × 10^6^ CAR^+^ T cells from either RNA-T cells (RNA-T 4D5-BBZ) or CD3/CD28 Dynabead T cells (Beads 4D5-BBZ) was given as a single injection. Mice treated with non-transduced RNA-T cells (RNA-T only) served as controls. Animals were imaged at the indicated time. (E) CD19 CAR lentiviral-transduced T cells generated by OKT3/IL-2 (OKT3), CD3/CD28 Dynabead (Beads) or OKT3-28BB/CD86/4-1BBL co-electroporated RNA (RNA-T) were compared in the Nalm6 leukemia model. Leukemia was established in NSG mice (*n* = 3 per group) by i.v. injection with 5 × 10^6^ Nalm6-CBG cells. Beginning on day 7, 1 × 10^6^ CD19-BBZ lentivirus-transduced T cells were injected (i.v.). BLI was conducted at the time indicated

RNA-T cells generated by co-electroporation of OKT3-28BB, CD86, and 4-1BBL were electroporated with RNA encoding an anti-CD19 bi-specific T cell engager (BiTE). The *in vivo* anti-tumor activity of these RNA-T cells was then tested in a mouse model of Nalm6 leukemia by giving a single dose (1 × 10^7^) of BiTE RNA-electroporated T cells, and the activity was compared with that of Dynabead T cells. A significant tumor reduction was observed for both the RNA-T cells and the Dynabead T cells after the mice were treated, and the tumors regressed to similar levels (Fig. 5A and 5B, *P* > 0.05). Both treatments significantly prolonged the survival of the treated mice compared with the control mice. Moreover, there was no significant difference in survival between the two treatment groups (Fig. 5C). To test the anti-tumor activity of the RNA-T cells in a solid tumor model, the T cells were transduced via a lentivirus with a 4D5-BBZ CAR against ErbB2. Unlike treating cancer using RNA-transferred T cells with transient anti-tumor activities, where multiple T cell infusions are needed to achieve long-term tumor repression, lentiviral transduction may provide T cells with sustained anti-tumor activities. RNA-T cells can be transduced with a lentiviral vector as efficiently as with CD3/CD28 Dynabead T cells (Fig. 6A). *In vitro* anti-tumor activities, such as CD107a up-regulation (Fig. 6B) and cytokine production (Fig. 6C), were similar between the lentivirus-transduced RNA-T and Dynabead T cells. In an ovarian tumor (SK-OV3) mouse model, CD3/CD28 Dynabead 4D5-BBZ CART cells showed faster tumor control than OKT3-28BB-86BBL RNA-T 4D5-BBZ CAR T cells. 4 out of 5 mice bearing large tumors (approximately 200 mm^3^ before treatment) were cured by 4D5-BBZ CAR-transduced OKT3-28BB-86BBL RNA-T cells, and 5 out of 5 were cured by CD3/CD28 Dynabead 4D5-BBZ CART cells (Fig. 6D). In our previous report comparing CD3/CD28 Dynabead T cells with OKT3/IL-2 T cells in the Nalm6 leukemia mouse model, the mice were treated with CD19 CAR RNA-electroporated T cells, and we found that OKT3/IL-2 T cells were inferior to the CD3/CD28 Dynabead T cells in controlling leukemia. Here, we lentiviral-transduced the same CD19 CAR into Dynabead T cells, OKT3-28BB-86BBL RNA-T cells, and OKT3/IL-2 T cells and tested their anti-leukemia activity in the Nalm6 model, and the OKT3/IL-2 T cells were still inferior to the CD3/CD28 Dynabead T cells in controlling leukemia. While the RNA-T CD19 CART cells were superior to the OKT3/IL-2 T cells in controlling leukemia, they were slightly inferior to the Dynabead CAR T cells (Fig. 6E).

## DISCUSSION

The culture of harvested lymphocytes with a soluble anti-CD3 antibody (OKT3) in the presence of interleukin (IL)-2 (Riddell and Greenberg, 1990) has been widely used and continues to be used in current cell therapy trials. Preliminary studies suggest that this method of expansion produces cells that are largely effector memory and effector T cells in phenotype (Gattinoni et al., 2005; Powell et al., 2005). Potential improvements have been explored, including the addition of feeder cells and the use of supplemental cytokines (Yang et al., 2010; Yang et al., 2013). In our current study, we sought to develop a novel T cell *ex vivo* expansion method that could easily be improved further. It has been reported that T cells can be stimulated and expanded by the K562 cell line expressing OKT3 scFv and other immune accessory molecules (Butler et al., 2012) and that synergy between CD28 and 4-1BB co-stimulation can be achieved by including their cytoplasmic domains arranged in tandem (Kloss et al., 2013; Stephan et al., 2007; Watts, 2005). In our study, an OKT3-28BB molecule was designed to combine stimulation (Anti-CD3 scFv is expressed on the cell surface to provide cis- and trans*-*stimulation to T cells) and co-stimulation (CD28 and 4-1BB, provided in cis form to T cells stimulated via anti-CD3 scFv) in a single structure for efficient human T cell expansion. It was found that T cells could only be expanded by starting with PBMCs, not with purified T cells (data not shown), suggesting that some cell components in the PBMCs essentially served as feeder cells to support efficient T cell expansion.

To develop a large number of T cells for adoptive immunotherapy, broad *ex vivo* cell expansion is required. Observations in murine tumor models and clinical trials have indicated that *in vivo* tumor treatment efficiency is mostly dependent on the differentiation status of the adoptively transferred T cells, where T cell differentiation is inversely related to *in vivo* anti-tumor effectiveness (Besser et al., 2010; Gattinoni et al., 2005). Thus, the use of “young”, less differentiated T cells with longer telomeres (Zhou et al., 2005b), high expression levels of CD27 and CD28 (Zhou et al., 2005a) and potent tumor lytic activities is crucial for success. In our previous work in a xenograft model of leukemia, we compared two T cell manufacturing methods that are commonly used in adoptive immunotherapy clinical trials, CD3/CD28 Dynabead and OKT3/IL-2. We found that CD3/CD28 Dynabead T cells mediated a greater anti-tumor response when mice were treated via intravenously infusion of CD19 CAR RNA-transferred T cells. This finding was consistent with our current findings that the CD19 CAR lentivirus-transduced CD3/CD28 Dynabead T cells mediate a greater anti-tumor response than do OKT3/IL-2 T cells in the same leukemia mouse model (Fig. 6E). However, compared with the CD3/CD28 Dynabead T cells, the OKT3/IL-2 T cells had a more heterogeneous phenotype and increased and sustained lytic activity *in vitro*. This finding suggests that the OKT3/IL-2 T cells can more potently induce tumor regression if the T cells are applied locally to the tumor sites, where less differentiated T cells with greater migration and proliferation ability are not as critically important as the T cells that are systematically infused. Upon intraperitoneally injecting T cells into mice to treat large pre-existing intraperitoneal human-derived tumors that had been growing *in vivo* for 56 days, we found a rapid and significant reduction in disease burden in mice receiving T cells expanded with OKT3/IL-2 (Fig. 2A and 2C). Significantly (10-fold) fewer T cells were detected in the peripheral blood of mice treated with mesothelin CAR-transferred OKT3/IL-2 T cells compared to mesothelin CAR-transferred CD3/CD28 Dynabead T cells (Fig. 2C). This result suggests that there was less leakage of OKT3/IL-2 T cells administered locally into the peripheral circulation due to their relatively high differentiation status and poor migration ability. This characteristic resulted in quick and improved local anti-tumor activities as well as the potential advantage of increased safety by avoiding unwanted off-target or on-target off-organ toxicities. Therefore, it might be important to consider the use of different T cell manufacturing procedures for treatments that require different T cell infusion routes. T cells expanded by RNA electroporation of OKT3-28BB are phenotypically and functionally similar to OKT3/IL-2 T cells and may be considered an alternative for treating cancers when local T cell administration is required. Instead of developing an alternative to pre-existing T cell manufacturing methods, the major goal of this study was to establish a stimulation and expansion platform upon which T cell quality could be easily improved to meet the required phenotypic characteristics, homing capacities, and potent effector functions for effective cancer immunotherapy (Rosenberg, 2008).

In addition to the direct use of tumor infiltrate lymphocytes (TILs) to treat cancer patients, T lymphocytes can be modified by gene transfer methods to permanently or transiently express therapeutic genes to enhance and expand their therapeutic potential (Maus et al., 2014). The requirements for transiently and permanently genetically modified T cells differ; immediate potent tumor killing ability and proper migration ability are more important for cells with transient gene transfers. By contrast, longevity, robust proliferative potential and the capacity to reconstitute the wide-ranging diversity of the T cell compartment are more critical for cells with permanent gene modifications. Extensive studies have focused on T memory stem cells (TSCM), defined as CD45RO^−^, CD45RA^+^, CD28^+^, CD27^+^, CCR7^+^, CD62L^+^, and IL-7Rα^+^ T cells, with increased levels of IL-2Rβ, LFA-1, CD95, and CXCR3; these studies have demonstrated several distinctive functions of memory cells. Compared with known memory populations, TSCM have shown increased proliferative capacity, greater efficiency in reconstituting immunodeficient hosts, and superior anti-tumor activities in animal models. TSCM identification is directly associated with vaccine design and T-cell-based therapies. TSCM can induce effective tumor regression when a limited number of cells are used. Tumor eradication may involve various components of the immune system. Thus, it is reasonable to transfer T cells to maintain a constant immunological attack against tumor masses. Therefore, a strategy that generates and expands TSCM-like cells is useful for the development of successful T-cell-based therapies (Gattinoni et al., 2011; Gattinoni et al., 2017). Human TSCM CAR T cells can be generated by CD3/CD28 stimulation with IL-7 and IL-15. CAR-modified CD8^+^ TSCM mediated superior and more durable anti-tumor responses than cells generated with protocols employed in clinical trials (Sabatino et al., 2016). One of the advantages of using RNA electroporation is that multiple splices of RNA can be introduced into T cells with high efficiency. Additionally, T cells can be genetically edited with high efficiency by electroporation of CRISPR/CAS9 (Ren et al., 2016). Thus, by co-introducing OKT3-28BB with RNA encoding other molecules, such as cytokines and co-stimulatory molecules, in combination with the CRIPSR gene editing of critical genes that regulate T cell differentiation, T cells with improved *in vivo* anti-tumor ability can ultimately be generated.

## MATERIALS AND METHODS

### Cell lines and primary human T lymphocyte cultures

The Nalm6 (DSMZ, Braunschweig, Germany), Raji (ATCC, Manassas, VA, USA), and K562 (ATCC, Manassas, VA, USA) cell lines were cultured per the suppliers’ instructions. CD19-expressing K562 cells and click beetle green (CBG)-expressing Nalm6 cells were generated as previously described (Barrett et al., 2014). SK-OV3, MCF7, MDA231, and MDA468 cell lines were purchased from the American Type Culture Collection (ATCC, Manassas, VA, USA) and cultured as instructed. PMBCs and purified primary lymphocytes from healthy donors were provided by the University of Pennsylvania Human Immunology Core. Primary T lymphocytes were stimulated and expanded using three different methods: 1. CD3/CD28 Dynabead (Life Technologies, Grand Island, NY) were used as previously described (Barrett et al., 2011). 2. For the OKT3/IL-2 approach, the obtained PBMCs were resuspended at a concentration of 2 × 10^6^/mL in culture medium supplemented with 50 ng/mL OKT3 and 300 IU/mL IL-2. The lymphocytes were then plated at 2 mL/well in 24-well plates (Costar). 3. For RNA-T cells, PBMCs were electroporated with OKT3-28BB RNA and re-suspended at a concentration of 2 × 10^6^/mL in culture medium supplemented with 300 IU/mL IL-2; the cells were then cultured and maintained following the same protocol for the OKT3/IL-2 T cells. The expanded T cells were aliquoted and frozen for further use. The T cells were cryopreserved in a solution of 90% fetal calf serum and 10% dimethylsulfoxide (DMSO) at 1 × 10^8^ cells/vial.

### Construction of vectors for RNA *in vitro* transcription (IVT), RNA IVT and electroporation

IVT vectors for CD19-BBZ, ss1-BBZ CARs, and 4D5-BBZ were constructed as previously described (Barrett et al., 2011; Liu et al., 2015). OKT3-28BB was constructed similarly to a CAR construct without the zeta cytoplasmic region, which includes the scFv from OKT3 (VL-linker-VH); the human CD28 hinge/transmembrane region; the CD28 cytoplasmic region; and the 4-1BB cytoplasmic region (Fig. S1). DNA encoding the blinatumomab BiTE (a CD19-CD3 bi-specific antibody) was synthesized based on published sequence data from patent US7575923. Human CD86 and 4-1BB cDNA were generated using RT-PCR with RNA isolated from activated T cells; the DNA was confirmed by sequencing. All genes were cloned into a pGEM.64A-based IVT vector (Zhao et al., 2003). The IVT vector was linearized by digestion with the appropriate restriction enzyme, and the mMESSAGE mMACHINE^®^ T7 Ultra kit (Life Technologies) was used to generate the IVT RNA according to the procedure provided with the kit. The frozen stimulated T cells were thawed and cultured overnight before electroporation. Prior to electroporation, the T cells were washed three times with OPTI-MEM and re-suspended in OPTI-MEM at a final concentration of 1–3 × 10^8^ cells/mL. Subsequently, 0.1 mL of the T cells was mixed with the indicated IVT RNA and electroporated in a 2-mm cuvette (Harvard Apparatus BTX, Holliston, MA) using an ECM830 Electro Square Wave Porator (Harvard Apparatus BTX) (Zhao et al., 2006).

### Flow cytometry analysis

Antibodies were obtained as follows: anti-human CD3 (BD Biosciences, 555335), anti-human CD8 (BD Biosciences, 555366), anti-human CD107a (BD Biosciences, 555801), and anti-human CD137 (BD Biosciences, 555956). The antibodies were incubated with T cells at 4°C for 25 min and washed twice (PBS with 2% FBS). Mesothelin CAR, ErbB2 CAR, CD19 CAR, and OKT3-28BB expression were detected by biotin-labeled polyclonal anti-mouse F(ab)2 antibody (Jackson Immunoresearch). Samples were then stained with phycoerythrin-labeled streptavidin (eBioscience, 17-4317-82). Flow cytometry acquisition was performed on either a BD FACSCalibur or Accuri C6 Cytometer (BD Biosciences). Analysis was performed using FlowJo software (Treestar).

### Enzyme-linked immunosorbent assay (ELISA)

Target cells were washed and suspended at 1 × 10^6^ cells/mL in R/10 (RPMI1640 with 10% FBS). One hundred thousand target cells of each type were added to each of 2 wells of a 96-well round bottom plate (Corning). Effector T cell cultures were washed and suspended at 1 × 10^6^ cells/mL in R/10. One hundred thousand effector T cells were combined with target cells in the indicated wells of the 96-well plate. Additionally, wells containing only T cells were prepared. The plates were incubated at 37°C for 18 to 24 h. After incubation, the supernatant was harvested and subjected to ELISA using standard methods (Pierce, Rockford, IL).

### CD107a assay

The cells were plated at an effector:target (E:T) cell ratio of 1:1 (10^5^ effectors:10^5^ targets) in 160 µL of R/10 medium in a 96-well plate. An anti-CD107a antibody was added and incubated with the cells for 1 h at 37°C before Golgi Stop was added and incubated for an additional 2.5 h. The anti-CD8 and anti-CD3 antibodies were added and incubated at 37°C for 30 min. After incubation, the samples were washed once and subjected to flow cytometry using a BD FACSCalibur. The data were analyzed by FlowJo software.

### Flow cytotoxic T lymphocyte (CTL) assay

A slightly modified version of a 4-h flow cytometry cytotoxicity assay was performed as previously described (Hermans et al., 2004; Zhao et al., 2010).

### Mouse xenograft studies

NSG mice were obtained from the Jackson Laboratory (Bar Harbor, ME) or bred in-house under an approved institutional animal care and use committee (IACUC) protocol and maintained under pathogen-free conditions. Six- to ten-week-old NOD-SCID-c^−/−^ (NSG) mice were bred in-house under an approved institutional animal care and use committee protocol. For the Nalm6 leukemia model, 1 × 10^6^ Nalm6-CBG cells (Nalm6 transduced via lentivirus with the click beetle green luciferase gene) were injected into each mouse via the tail vein. The T cells were injected via the tail vein either 5 or 7 days after the Nalm6-CBG cells were injected, as indicated. Tumor growth was monitored using bioluminescence imaging (BLI) as previously described (Barrett et al., 2011). For the M108 mesothelioma model (Carpenito et al., 2009; Zhao et al., 2010), animals received intraperitoneal injections with 8 × 10^6^ viable M108-Luc. Tumor growth was monitored using BLI every two weeks for 4 weeks after the tumor was injected. T cells were injected intraperitoneally 8 weeks after tumor inoculation. Tumor growth was monitored using BLI every week after T cell injection. For the SK-OV3 ovarian cancer model, studies were performed as previously described with certain modifications (Liu et al., 2015). Briefly, 6- to 10-week-old NOD-SCID-γ^−/−^ (NSG) mice were subcutaneously injected with 5 × 10^6^ SK-OV3-CBG tumor cells in the right flank on day 0. The mice were treated with T cells via the tail vein on day 18 post-tumor inoculation, when the tumors were approximately 200 mm^3^ in volume.

### Bioluminescence imaging

Anesthetized mice were imaged using a Xenogen Spectrum system and Living Image v3.2 software. The mice were given an intraperitoneal injection of 10 mg/kg body weight D-luciferin (Caliper Life Sciences, Hopkinton, MA) suspended in sterile PBS at a concentration of 15 mg/mL (100 μL luciferin solution/10 g mouse body weight). Previous titration of both Nalm6 and human T cells transduced with the firefly luciferase vector revealed a time to peak of photon emission of five minutes, with peak emission lasting 6–10 min. Each animal was imaged alone (for photon quantitation) or in groups of up to 5 mice (for display purposes) in the anterior-posterior prone position at the same relative time point after luciferin injection (6 min). Data were collected until the mid-range of the linear scale was reached (600 to 60,000 counts) or until maximal exposure settings were reached (f stop 1, large binning and 120 s) and were then converted to photons/s/cm^2^/steradian to normalize each image for exposure time, f stop, binning and animal size. For anatomic localization, a pseudocolor map representing light intensity was superimposed over the grayscale body-surface reference image. For data display purposes, mice without luciferase-containing cells were imaged at maximal settings, and a mean value of 3.6 × 10^5^ p/s/cm^2^/sr was obtained. Mice with luciferase-containing Nalm6 typically became moribund with leukemia when the photon flux approached 5 × 10^11^ p/s/cm^2^/sr, giving a detection range of 6 orders of magnitude.

### Statistical considerations

Analyses were performed using STATA version 10 (StataCorp, College Station, Texas) or Prism 4 (Graphpad Software, La Jolla, CA). The *in vitro* data represent the mean of duplicates, and comparisons of means were performed using the Mann-Whitney test. For comparisons among multiple groups, Kruskal-Wallis analyses was performed with Dunn multiple comparison tests to compare individual groups. The leukemia burdens, as measured by BLI of the different groups, were compared with the Mann-Whitney test. The Student’s *t*-test was performed to compare differences in T cell proliferation.

## Electronic supplementary material

Below is the link to the electronic supplementary material.

Supplementary material 1 (PDF 580 kb)
