# Supplementary material for: Novel T cells with improved in vivo anti-tumor activity generated by RNA electroporation
Source: Protein Cell. 2017 May 18;8(7):514–26. doi: 10.1007/s13238-017-0422-6 (PMC5498341; doi:10.1007/s13238-017-0422-6)
Supplement: Supplementary file 1 — Supplementary material 1 (PDF 580 kb) [file 13238_2017_422_MOESM1_ESM.pdf]

Supplementary data

Novel T cells with improved in vivo anti-tumor activity generated by RNA electroporation

Xiaojun Liu[1,2], Shuguang Jiang[2], Chongyun Fang[2], Hua Li[2], Xuhua Zhang[2], Fuqin Zhang[2], Carl H. June[2, 3] and Yangbing Zhao[2,3]

Running title: Generating novel T cells by RNA electroporation to improve cancer treatment

Keywords: T lymphocytes, CAR, manufacture, gene transfer, RNA electroporation

* To whom correspondence should be addressed:

Yangbing Zhao, M.D., Ph.D.
University of Pennsylvania School of Medicine
Center for Cellular Immunotherapies
3400 Civic Center Blvd TRC 421, RM08-122
Philadelphia, PA 19104-5156
Email: Yangbing@upenn.edu

[1] Key Laboratory for Experimental Teratology of Ministry of Education and Department of Immunology, School of Basic Medical Sciences, Shandong University, Jinan, China

[2] Center for Cellular Immunotherapies, University of Pennsylvania Cancer Center, Philadelphia, PA 19104

[3] Department of Pathology and Laboratory Medicine, Perelman School of Medicine, University of Pennsylvania, Philadelphia, Pennsylvania

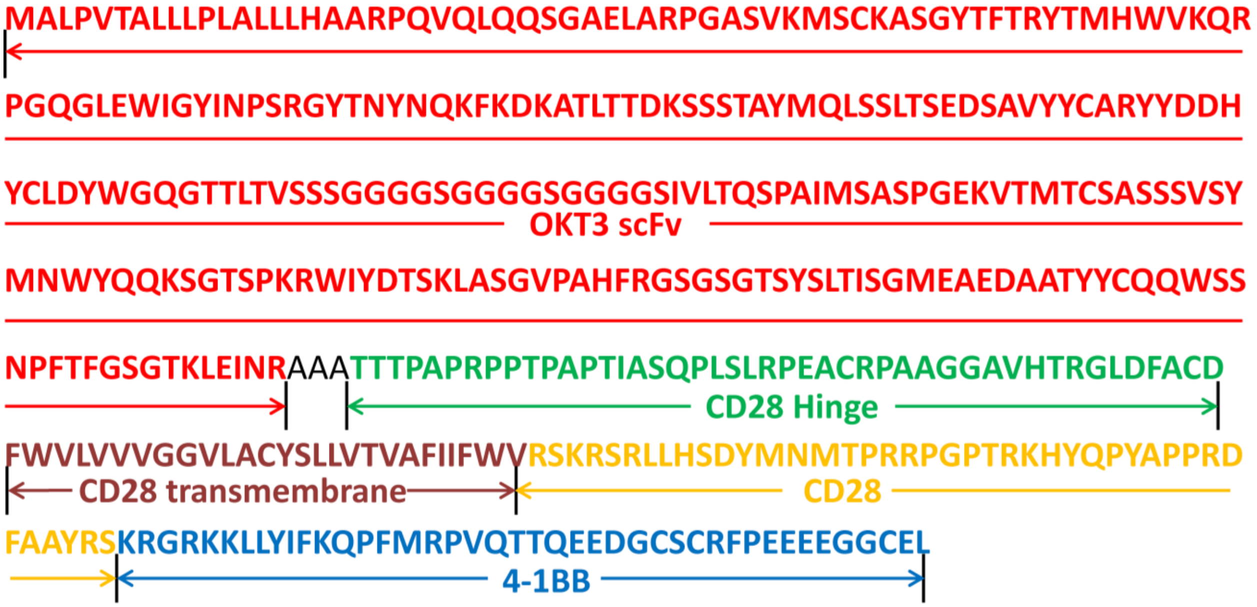

Supplemental Figure 1. Sequence of OKT3-28BB. The sequence is composed of following parts as indicated: OKT3 Single-chain variable fragment (OKT3 scFv), hinge from CD28 (CD28 Hinge), transmembrane from CD28 (CD28 transmembrane), cytoplasmic region of human CD28 (CD28) and cytoplasmic region of human 4-1BB (4-1BB).

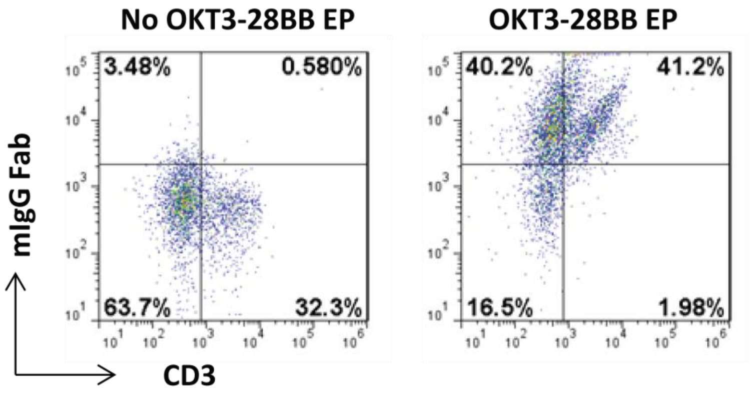

Supplementary Figure 2. OKT3-28BB expression of RNA electroporated PBMCs. PBMCs from a normal donor were electroporated with OKT3-28BB RNA (OKT3-28BB EP) and OKT3-28BB expression was detected using an antibody against murine IgG Fab (mIgG Fab) 18h post RNA electroporation. PBMCs that were not electroporated with OKT3-28BB RNA (No OKT3-28BB EP) was used as control.

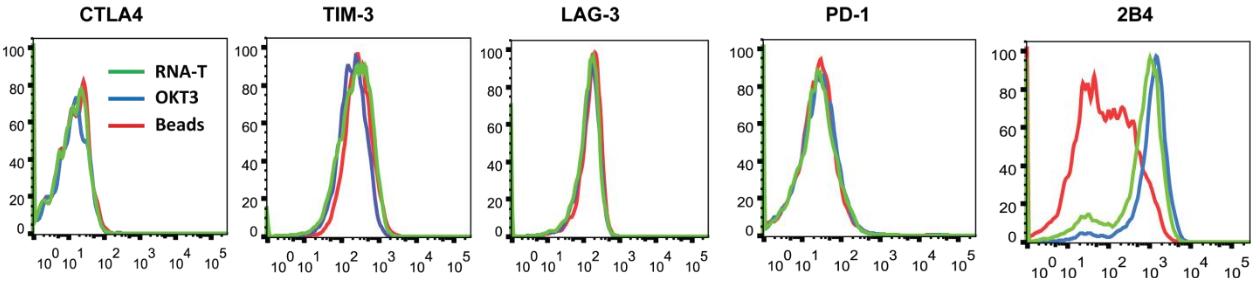

Supplementary Figure 3. Expression of exhaustion markers. Expression of the exhaustion markers CTLA4, TIM-3, LAG-3, PD-1 and 2B4 were detected respectively for the T cells generated by OKT3-28BBZ RNA electroporation (RNA-T), OKT3/IL-2 stimulation (OKT3) or CD3/CD28 DynaBead stimulation (Beads) 10 days after stimulation.
